# Photo-Polymerization in Chiral Dopant Liquid Crystal Cells via Holographic Exposure to Fabricate Polarization-Independent Phase Modulator with Fast Optical Response

**DOI:** 10.3390/polym10030315

**Published:** 2018-03-14

**Authors:** Chun-Yu Chien, Pin-Kuan Sung, Chia-Rong Sheu

**Affiliations:** Department of Photonics, National Cheng Kung University, Tainan 70101, Taiwan; L78031033@mail.ncku.edu.tw (C.-Y.C.); L76061163@mail.ncku.edu.tw (P.-K.S.)

**Keywords:** chiral dopant, liquid crystals, polarization-independence, phase modulator

## Abstract

Small liquid crystal domains with random director distributions were obtained to show novel optical isotropy using a holographic exposure processes to treat chiral dopant liquid crystal cells in the isotropic phase (i.e., polymer-stabilized isotropic liquid crystal cells). The cells used to fabricate phase modulators showed unique performances, including low light scattering, polarization-independence, and fast optical response. Furthermore, an extra fluoro-surfactant dopant in cells showed that the phase modulators retained their performance but with considerable reduction of operating voltages, from 180 V_rms_ to 100 V_rms_.

## 1. Introduction

Optical birefringence and dielectric anisotropy in liquid crystals (LCs) play key roles in making various electric-optical devices that are convenient to use, cheap, and easily fabricated. Most LC devices are used to modulate optical intensity and/or phase modulation based on electrically controllable LC-reorientation with respect to various alignments for real applications [1,2,3,4,5,6]. For example, the homogeneous alignment LC (HALC) cell is usually considered and used as a basic type of phase modulation device for laser beam steering [4], spatial light modulators [5], and tunable focus lenses [6]. However, larger cell gaps are usually needed in HALC cells to fit optimal phase modulations. As a result, the performance of the optical response time will be significantly degraded due to large cell gaps. Simultaneously, most LC devices are polarization-dependent. Basically, it is not very clear whether the better choice of electro-optical LC devices is polarization-dependent or polarization-independent. However, polarization-independent LC devices usually achieve operation convenience in most optical systems. To improve optical response time, a method named polymer network LC (PNLC) was explored in previous studies using photo-curable anisotropic monomers in cells [7,8,9,10,11]. Anisotropic monomers with similar structures to LC building blocks along with LC molecules are usually well aligned by means of LC alignment layers. After photo exposure processes, the generated polymer networks provide a constrained force for LC molecules to speed up reorientation time during electrical operation. In addition, some research studies have demonstrated various electro-optical devices that are based on LC/polymer composited cells, which have achieved improved optical response times [12,13]. However, these devices are also polarization-dependent.

Apart from the improved response time, some research studies have emphasized that the optical performance of fabricated devices is dependent on the type of monomer structures used and the fabrication process [14,15,16,17,18,19]. By using isotropic monomers to fabricate polymer dispersed LC (PDLC) cells for applications such as light shutters and switchable windows [18,19], it is usually shown that they possess optical polarization-independence. In PDLC cells, dense LC droplets with averaged micro-meter dimensions are randomly dispersed in the polymer matrices. When an incident light beam passes through the PDLC cell, random optical paths of the incident light beam are observed due to significant local mismatch of refractive indices between polymer matrices and LC droplets. In regard to the available phase modulators based on PDLC cells with lower light scattering and polarization-independence, it is necessary that the fabricated devices have much smaller LC droplet dimensions and be electrically operated with higher voltages [20,21]. Even if this holds true, available PDLC phase modulation is not large enough for real applications. In contrast, anisotropic monomers are also available for fabricating polarization-independent phase modulators in homeotropic PNLC cells [22], polymer stabilized cholesteric LC cells [23], and polymer stabilized short-pitch planar cholesteric LC cells [24]. Polarization-independent LC phase modulators with anisotropic monomer dopant usually possess a larger phase modulation, but higher voltages are also needed during operation.

In addition to the previously stated research, H. Kikuchi et al. proposed a method using polymer stabilized blue phase LC (PSBPLC) to expand blue phase LCs with a larger temperature range to fit the requirements of real applications [25]. After that, various devices based on PSBPLC cells were demonstrated, such as phase modulators, lenses, and displays, which possess many unique characteristics, including optical isotropy, non-LC alignments, and sub-millisecond response times with an electric Kerr effect [26,27,28,29]. Numerous approaches were continuously investigated, along with an improved electrical-optical performance of PSBPLC, such as phase retardation and Kerr constant [30,31,32]. Similar to PSBPLC, Y. Haseba et al. found an interesting formation of optically isotropic LC via photo-polymerization in the polymer/chiral LC mixture cell at 2 K above the isotropic phase (i.e., transition temperature or cleaning point *T_c_*) [33,34], which was named polymer-stabilized isotropic LC (PSILC) cells. Recently, Y. C. Yang et al. demonstrated a similar photo-polymerization in PSILC cells at much higher temperatures above *T_c_* [35]. According to their illustrations in the reference, growth directions of generated polymer networks do not seem to be affected by original LC alignments because photo-polymerization occurs in the isotropic LC phase. Furthermore, the chiral dopant was used to further randomize the LC orientation, to achieve much smaller dimensions of multi-LC domains with significant optical isotropy.

Holographic exposure is a usual process to treat photo-curable monomer dopant LC cells to fabricate switchable optical gratings and filters via phase separation procedures. The generated optical interference patterns eventually correspond to the formation of periodic layer structures with extremely small pitches in LC cells. Two major types of holographic gratings are usually demonstrated in research, one of which is holographic polymer dispersed LCs (HPDLCs) and the other is polymer liquid crystal polymer slices (POLICRYPS). The former shows typical structures composed of alternating nanometer-dimension LC droplets and cross-linked polymers in cells, which possesses the characteristics of easy fabrication, fast optical response, and good optical performance [36,37,38]. However, light scattering issues and higher operating voltages usually occur to limit their applications. In contrast, the latter processed with holographic exposure at a high temperature leads to more completed structures of phase separation between LCs and polymer, so that lower operating voltages and low scattering loss are obtained [39,40]. In addition to optical gratings, R. Caputo et al. proposed a particular POLICRYPS to determine the various electric-optical devices with low operating voltages, including phase modulators, optical micro-resonators, and tunable optical filters [41]. Instead of nematic LCs, E. R. Beckel et al. improved the electro-optical performance in polymer stabilized cholesteric LC (PSCLC) cells via holographic exposure processes to show characteristics of higher transmittance and lower operating voltages [42]. In our previous work, holographic exposure processes with low-power He–Ne lasers were used to treat HALC cells with anisotropic monomer dopant using the smallest pitch of interference patterns—about 200 nm—to successfully generate much smaller dimensions of LC domains. The fabricated polarization-dependent PNLC phase modulator showed good electro-optical performance with an ultra-fast optical response time and low light scattering [9]. In the present study, the holographic exposure processes were used again to treat the polymer/chiral dopant LC mixture in isotropic phase to achieve polarization-independent phase modulation. As a result, HALC cells doped with a low concentration of an anisotropic monomer were processed as PSILC cells with non-holographic exposure processes to show the fabricated sample with significant light scattering phenomena. When using holographic exposure processes, small interference pitches provide more constraints to generate smaller dimensions of LC domains to reduce light scattering issues. Simultaneously, the chiral dopant provides the potential to further randomize LC directions to increase transmittance in cells. The performance of PSILC phase modulators were investigated and improved via holographic exposure processes, including transmittance, phase modulation versus polarization directions, optical response time, and scanning electron microscopy (SEM) analysis of PSILC cells with/without chiral dopant. Finally, a fluoro-surfactant dopant in PSILC was used to reduce the constraints of polymer networks to reduce operation voltages.

## 2. Experimental

### 2.1. Materials

**Figure 1:**
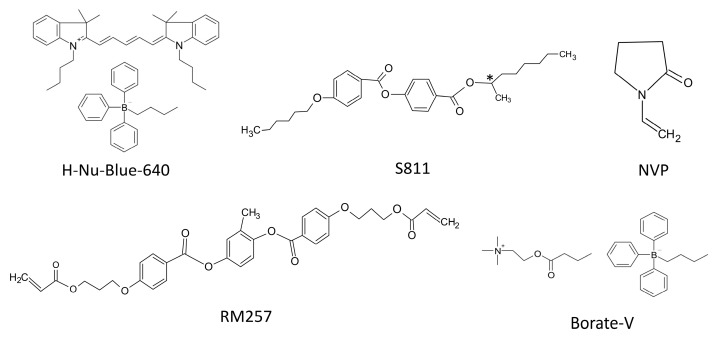
Chemical structures of all ingredients in the prepared liquid crystal (LC) mixture except for nematic LCs.

First, the LC mixture used to fill in cells was prepared. This included nematic LCs (HTG135200-100, HCCH, Yangzhong, China), chiral dopant (S811, Fusol Material, Tainan, Taiwan), anisotropic monomer (RM257, HCCH, Yangzhong, China), mono-acrylate monomer (NVP, Sigma-Aldrich, St. Louis, MO, USA), photo-initiator (H-Nu-Blue-640, Spectra Group Limited, Millbury, OH, USA), and co-initiator (Borate-V, Spectra Group Limited, Millbury, OH, USA). Figure 1 individually indicates chemical structure of all above-mentioned ingredients, except for nematic HTG LCs. The physical parameters of nematic HTG LCs at 20 °C were as follows: extraordinary refractive index, *n_e_* = 1.717, and ordinary refractive index, *n_o_* = 1.513, at 589 nm visible wavelength (i.e., optical birefringence *Δn* = 0.204); dielectric anisotropy *Δε* = 57.2 at 1 kHz electric signal frequency; viscosity, 183 mm^2^·s^−1^; and transition temperature, (*T_c_*), 97 °C. The anisotropic monomer, RM257, which has similar building block to LC molecules shows the LC phase at a temperature range of 70–126 °C, with completed morphology of polymer networks and random growth directions to constrain LCs after the process of photo-polymerization in the isotropic phase. H-Nu-Blue-640 and Borate-V were used to initiate the beginning of photo-polymerization via holographic exposure processes. Rather than using NVP dopant to optimize the electro-optical performance in PNLC cells [43], NVP was mainly utilized as the homogenizer to mix H-Nu-Blue-640 and Borate-V to yield a uniform precursor. Finally, LC mixture was completed by combining all ingredients and continuously stirring in the isotropic phase for 30 min. Then, the LC mixture was used to fill a commercialized empty LC cell (homogeneous LC alignment with 15 μm cell gap, purchased from Chipset Technology, Miaoli, Taiwan) via the capillarity effect. Table 1 shows a list of experimental cells with various ratios of ingredients of LC mixtures for processing holographic exposure. The first five cells with fixed ratios of H-Nu-Blue-640, Borate-V, RM257, and NVP were investigated in regard to the optical performance of PSILCs with respect to various ratios of HTG LCs versus S811 ingredients. The final cell was investigated in regard to the reduction of electric operation voltages via the extra addition of 2 wt % fluoro-surfactant (FS3100, DuPont, Wilmington, DE, USA) instead of the original ratio of HTG LCs, to possibly reduce constraints between LCs and polymer networks.

### 2.2. Holographic Exposure Setup

**Figure 2:**
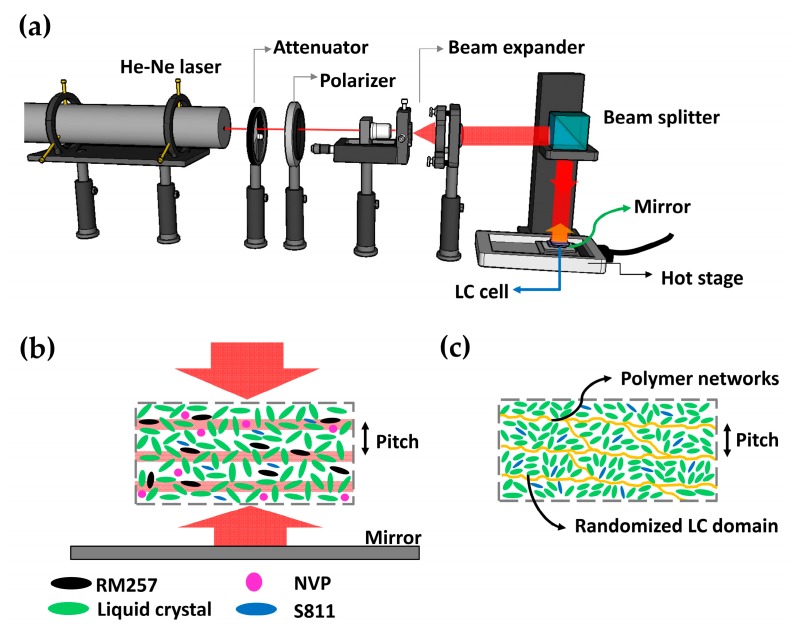
(**a**) Schematic setup and holographic exposure processes; (**b**) Schematic location of LC layer in the cell with holographic interference inside for the exposure process in the isotropic phase; (**c**) After exposure processes, there are small LC domains with random LC director distributions around the polymer networks.

In general, holographic exposure techniques are categorized into two types, one of which is amplitude type and the other is polarization type. The former uses two laser beams with the same linear polarization to achieve optical interference patterns to process the cells [37,39]. In contrast, the latter uses two laser beams with orthogonal polarizations to achieve spatial polarization modulation, to process the cells [38,44]. In the present study, the former type of holographic exposure was adopted to process the LC cells. The setup used to process holographic exposure in cells at above isotropic phase temperature is shown in Figure 2a. A He–Ne laser beam (*λ* = 632.8 nm, LASOS, Jena, Germany) continuously passing through the attenuator, polarizer, and beam expander finally achieved an expanded and collimated s-polarization light beam. This beam was reflected via a beam splitter to further expose the experimental LC cell to incident light. In order to achieve holographic exposure and isotropic phase in cells, a hot stage with temperature controller (model: T96-P, LINKAM, Tadworth, UK) and a plane mirror were placed under the experimental LC cell. Meanwhile, a refractive index liquid (*n* = 1.524, Cargille Labs, New York, NY, USA) was filled into the gap between the LC cell and plane mirror to prevent optical interference issues. Figure 2b schematically shows holographic exposure processes in the cell by means of normal incident and reflective coherent laser beams. The power intensity of the incident light beam was 50 μW/cm^2^ to facilitate holographic exposure for 1 h. Both laser beams with s-polarization generated optical interference with a crossed angle of 2*θ* = 180°, so that the theoretical pitch (Λ) of the interference pattern could be determined with Equation (1) [45]. In Equation (1), the parameter *n_eff_* is the effective refractive index of the LC mixture in the isotropic phase, which can be calculated as *n_eff_ ≈* (2*n_o_ + n_e_*)/3; a value of 200 nm was obtained in this study.

(1) $$\Lambda =\frac{\lambda }{2n_{eff}\sin\theta }$$

In general, the ratios of chiral dopant in LC cells are very sensitive to LC orientation distributions and directly affect electro-optical properties [46]. In this study, the cell was in the isotropic phase at a fixed temperature of 120 °C above the *T_c_* of LC mixtures that are exposed with holographic exposure processes. During photo exposure processes, the non-uniform light irradiation in the LC cell possibly occurred due to the optical absorption of LC mixtures which generates the formation of gradient distribution in polymer networks. This issue can be depressed by using a symmetrical configuration of light beams to expose the cell [47,48,49]. When processing photo exposure, anisotropic monomer RM257 and isotropic NVP monomer moved toward the regions of higher intensity (i.e., constructive interference areas) to generate photo-polymerization, so that the gradient distribution of polymer networks was not necessarily formed in cells. Simultaneously, chiral dopant will possibly cooperate with monomers and LCs to generate much smaller LC domains with random LC director distributions, causing optical isotropy and reducing light scattering issues, as schematically shown in Figure 2c.

### 2.3. Measurement

The PSILC cells, shown in Table 1, were prepared to investigate their electro-optical performance after photo exposure processes. The optical transmittance spectra in completely exposed PSILC cells with various ratios of chiral dopant S811 were measured by means of an unpolarized white light source (model: SL1, StellarNet, Tampa, FL, USA) and a fiber spectrometer (model: Blue-Wave, StellarNet, Tampa, FL, USA). Normalized transmittance spectra were determined by the ratio of values with respect to the transmittance spectrum of only HTG LC filled cells. In addition, morphology variations of generated polymer networks were recorded and analyzed via SEM (model: JSM-6340F, JEOL, Tokyo, Japan), with samples prepared by cell decomposition and immersion in *n*-hexane solution for 1 h.

A setup of the Mach–Zehnder interferometer was used to measure electric phase modulation in completed PSILC cells. In this setup, an unpolarized He–Ne laser beam (λ = 632.8 nm, LASOS, Jena, Germany) was expanded and collimated to be incident to a beam splitter in order to separately output two laser beams with coherence. The measured PSILC cell was located in one path of laser beams. Finally, two laser beams, one passing, and one not passing the PSILC cell were reflected by individual mirrors and re-combined by another beam splitter to achieve optical interference. A charge-coupled device (CCD) camera was used to record digital images of interference patterns. When measuring phase modulation in PSILC cells, it was applied with various AC voltages using a 1 kHz square waveform from both a function generator (model: 33220A, Agilent, Santa Clara, CA, USA) and a power amplifier (model: F10A, FLC Electronics, Partille, Sweden). Finally, the performance of the optical response time in completed PSILC cells was measured by means of Mach–Zehnder interferometer; however, a photoreceiver (model: 2001-FS, New Focus, Santa Clara, CA, USA) was used instead of the CCD camera. In order to achieve measurements with convenience, a lens and an iris were used to magnify the interference patterns to only allow intensity variations of dark fringe to be recorded by the photoreceiver.

## 3. Experimental Results

**Figure 3:**
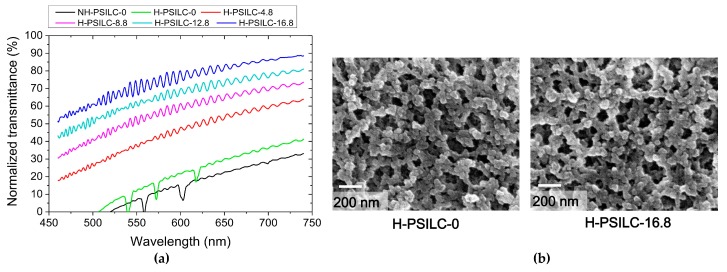
(**a**) Comparisons of normalized transmittance spectra in PSILC cells with various ratios of the ingredients listed in Table 1. Both NH-PSILC-0 and H-PSILC-0 cells without S811 chiral dopant were compared in terms of transmittance spectra with individual non-holographic and holographic exposure processes; (**b**) Comparisons of polymer network morphology by SEM images in both PSILC cells with and without S811 chiral dopant.

**Figure 4:**
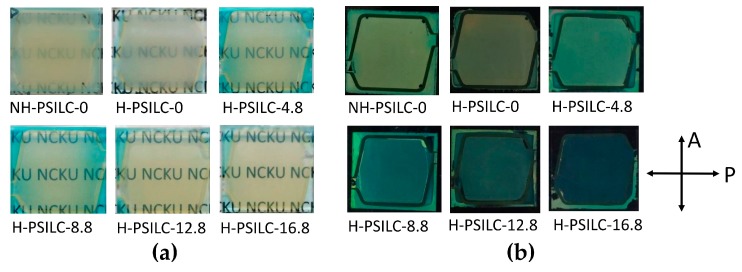
Comparisons of optical observations in different PSILC cells: (**a**) Images of “NCKU” characters. Non-exposed areas show blue color due to the existence of non-reactive photo-initiators; (**b**) The degrees of dark states in cells located between a pair of crossed polarizers.

First, it must be demonstrated and emphasized that significantly improved transmittance spectra are achieved in cells processed with holographic exposure. As shown in Figure 2a, the plane mirror is removed when processing in a non-holographic exposure, and the incident optical power intensity has double values (i.e., 100 μW/cm^2^) compared to holographic exposure processes for 1 h. Cells with the same LC mixture and no chiral dopant S811 were individually processed via holographic and non-holographic exposures, with the former labeled as H-PSILC-0 and the latter cell labeled as NH-PSILC-0. Figure 3a shows comparisons of normalized transmittance spectra with respect to completed cells with various ratios of ingredients. In general, morphology types of generated polymer networks as anisotropic monomers in cells are usually sensitive to cell conditions during photo-exposure processes, including cell temperature [50,51,52], light intensity [50], solubility of monomers and LC mixtures [53,54], LC alignments [55], and various types of LC phase [56]. Therefore, when photo processing in LC cells at higher temperatures, faster reaction of phase separation occur due to the lower LC viscosity, so more polymer fibrils link to form larger polymer bundles and correspondingly larger LC domains. In particular, the morphology of polymer networks usually shows a significant difference when the processing temperature is close to the isotropic phase temperature, *T_c_*. At temperatures below *T_c_*, polymer network morphology usually possesses a unidirectional growth direction for fibrous combinations, which follow the initial LC alignments [50,53]. As a result, completed cells maintain their homogenous alignment and show transparence, but light scattering issues are significant due to the appearance of micro-meter dimensions of LC domains during electrical operation. In contrast, the polymer network morphology shows bead-like shapes without a special growth direction at temperatures higher than *T_c_*, so that the completed LC cells show serious light scattering issues due to larger LC domains and random LC director distributions [51,55]. Figure 3a shows the difference between normalized transmittance in cells without chiral dopant S811 and individually processed non-holographic and holographic exposure processes. One cell, labeled NH-PSILC-0, possessed the lowest transmittance spectrum, and the other cell, labeled as H-PSILC-0, possessed a slightly higher transmittance spectrum than NH-PSILC-0. This means that smaller dimensions of LC domains are surely achieved via holographic exposure processes which reduce light scattering issues. However, the improved transmittance is not large enough. In order to achieve higher transmittance improvements, a chiral dopant S811 is added, and instead of original percentages of LCs in cells, S811 provides an effect that further randomizes the LC director distributions, producing much smaller multi-LC domains. Higher transmittance spectra are obviously proportional to percentages of chiral dopant S811 in cells with holographic exposure processes. Figure 3b shows SEM image comparisons of the polymer network morphology that occurred on the locations of cell substrates of PSILC cells with and without S811 chiral dopant. Some similarities are obvious, including the dimensions and shapes of the polymer network morphology, and the occurrence of polymer networks stacked in numerous bead-like polymer units. Thus, the chiral dopant S811 facilitated the randomization of LC director distributions. Figure 4a shows comparisons of light scattering issues in different PSILC cells. The cells were placed away in a fixed distance over an object with “NCKU” characters on it. Without chiral dopant in the cells, H-PSILC-0 cells show a clearer image of the “NCKU” characters than NH-PSILC-0, due to improvements in light scattering issues following the holographic exposure process. Furthermore, the clearer images of the “NCKU” characters are proportional to the percentage of chiral dopant S811 used when processing cells with holographic exposure. The completed cells were also located between a pair of crossed polarizers to observe whether dark states are related to optical polarization or not (Figure 4b). If the cells show a very dark state, it means that more optical isotropy and lower light scattering are achieved. Obviously, the H-PSILC-16.8 cell belongs to the cell with optical isotropy and low light scattering. In order to further improve the dark states of cells, it is possible to add a co-polymer dopant such as NVP material in an optimal concentration [11,43].

**Figure 5:**
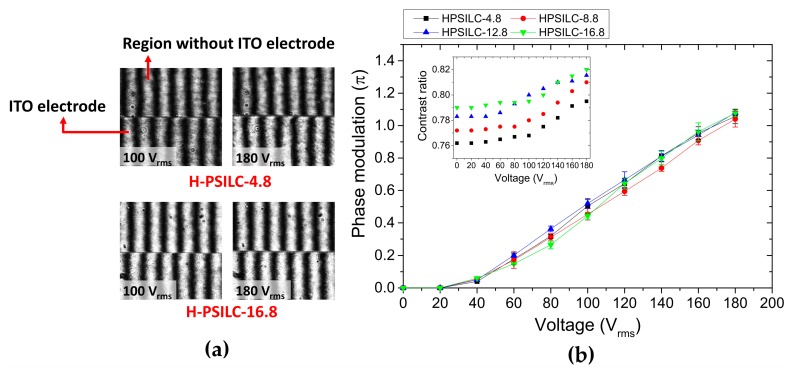
(**a**) Shift of interference patterns occurring in the boundary with or without ITO electrode in both H-PSILC-4.8 and H-PSILC-16.8 cells with applied voltages of 100 and 180 V_rms_; (**b**) Electric phase modulation in PSILC cells with various percentages of S811 chiral dopant. Insert shows *CR* values with respect to the applied voltages.

Figure 5a shows phase modulation via the interference pattern variations in PSILC cells labeled as H-PSILC-4.8 and H-PSILC-16.8, following individually applied voltages of 100 and 180 V_rms_. The shift of interference patterns occurred in the boundary where the interface, either with and without the indium tin oxide (ITO) electrode in the cell. Thereafter, optical phase modulation can be evaluated by means of the shift of interference patterns with respect to variously applied voltages, as shown in Figure 5b. Performances of phase modulations are obviously very consistent in the different PSILC cells with various percentages of S811 chiral dopant. The maximum applied voltage was controlled at 180 V_rms_ in order to achieve only about 1.1 π phase modulation due to the possibility of dielectric breakdown damage, which can occur in LC cells as the applied voltage gets higher. In addition, the contrast ratio (*CR*) of interference patterns can also be evaluated using the equation, *CR =* (*I_max_ − I_min_*)/(*I_max_ + I_min_*), where *I_max_* and *I_min_* are the maximum and minimum intensities of interference patterns recorded by the CCD camera (Figure 5b). At electric off states, *CR* values in PSILC cells with 4.8, 8.8, 12.8, and 16.8 wt % S811 chiral dopant had values of 0.762, 0.772, 0.783, and 0.79, respectively. A lower *CR* is attributed to larger light scattering in interference patterns, as shown in the H-PSILC-4.8 cell in Figure 5a. With increasing applied voltages, CR values also increased proportionally to the applied voltages in PSILC cells. At a voltage of 180 V_rms_, *CR* values in PSILC cells with 4.8, 8.8, 12.8, and 16.8 wt % S811 chiral dopant had values of 0.795, 0.811, 0.815, and 0.821, respectively.

**Figure 6:**
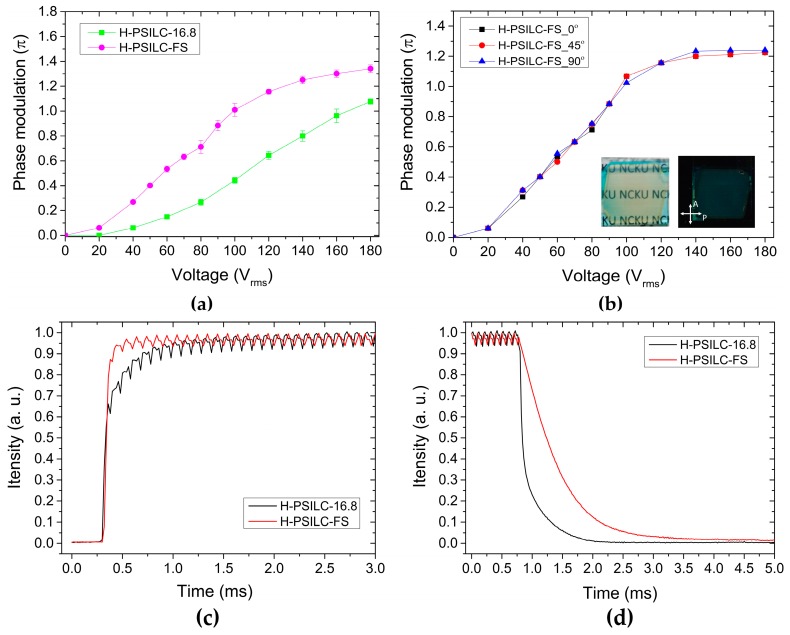
Comparisons of the electro-optical performances in H-PSILC-16.8 and H-PSILC-FS cells: (**a**) Electric phase modulation; (**b**) Electric phase modulation with respect to various incident linear polarizations in H-PSILC-FS cells. Inserts show the transparent and off (dark) states observed with a pair of crossed polarizers; (**c**) Measurements of transient rising time; (**d**) Measurements of transient falling time.

Finally, a dopant of FS3100 fluoro-surfactant (2 wt %) instead of partly original percentages of HTG LCs was added into the H-PSILC-16.8 cell to try to reduce the operation voltages with cells labeled as H-PSILC-FS. Figure 6a shows the measured results of electric phase modulation in H-PSILC-16.8 and H-PSILC-FS cells. Obviously, H-PSILC-FS cell showed a π phase modulation with an applied voltage of 100 V_rms_. This value is lower than the H-PSILC-16.8 cell with a voltage of 180 V_rms_ at the same phase modulation. It is possible that FS3100 dopant is used to reduce constraints between polymer networks and LC molecules so that LC reorientation is easier with respect to electric operation. Aside from the applied voltage reduction, the electro-optical performance in the H-PSILC-FS cell was very consistent and as good as the performance in the H-PSILC-16.8 cell. Figure 6b shows a good dark image in the off state and a high transparent image in the on state, as observed with a pair of crossed polarizers. Simultaneously, significant polarization-independent electric phase modulation is also achieved with respect to various incident linear polarizations of 0°, 45°, and 90°.

Figure 6c,d show the optical response times of π phase modulation in H-PSILC-16.8 and H-PSILC-FS cells. To determine this, measured cells were directly electrically switched between on and off states of π phase modulation with 10 kHz AC voltages in order to prevent the occurrence of a large phase ripple [57]. When processing a π phase modulation with a 10 kHz AC voltage (square waveform) in PSILC cells, the initial dark fringe becomes a brightness fringe due to the shift of interference patterns. The rising time (*τ_r_*) is defined as the time spent changing intensity variations from 0 to 90% and the falling time (*τ_f_*) represents the time spent changing intensity variations from 100% to 10%. As a result, the H-PSILC-16.8 shows *τ_r_* = 0.44 ms and *τ_f_* = 0.54 ms when switching electrically between 0 and 180 V_rms_. H-PSILC-FS shows *τ_r_* = 0.11 ms and *τ_f_ =* 1.32 ms when switching electrically between 0 and 100 V_rms_. In general, the optical response time in mixed LC/polymer cells is very sensitive to the dimensions of LC domains, LC elastic constants, and LC rotational viscosity. In addition, the rising time is also sensitive to the applied voltages in the cells [58,59]. Due to the consistent dimensions of LC domains in H-PSILC-16.8 and H-PSILC-FS cells according to their similar transmittance in cell images, H-PSILC-FS cells show a slower falling time (Figure 6d) that attributed to the constraints between polymer networks and LCs are reduced as a consequence of the FS3100 dopant. Furthermore, this effect speeds up the rising time because of an easier LC orientation with respect to the applied electrical fields.

## 4. Discussion

**Figure 7:**
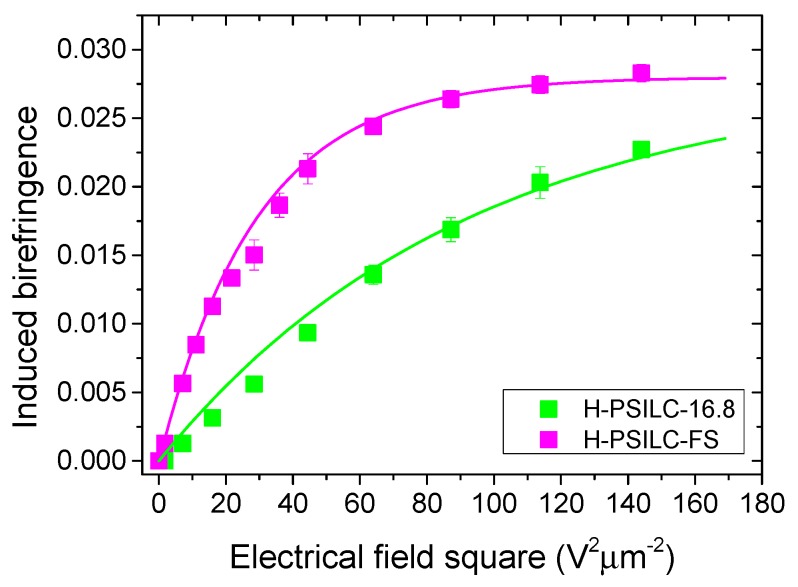
The induced birefringence in PSILC cells and correspondence of the fitting curves, in accordance with Equation (2).

When applying electric voltages in cells with optically isotropic LCs, an electrical field induced birefringence usually occurs via the Kerr effect and follows the equation, *Δn = λ∙K∙E^2^*, in a small electrical field region. J. Yan et al. proposed an extended Kerr effect [60], which follows Equation (2) to get a correct predicted trend of the induced birefringence (Δ*n_ind_*). The induced birefringence will increase from zero to the maximum birefringence (Δ*n_max_*) as the electrical field exceeds the saturating electrical field (*E_sat_*). In accordance with the theory of the extended Kerr effect, experimental results in PSILC cells were analyzed and investigated based on their correspondence to the Kerr constant. The experimental data shown in Figure 6a were converted into relative curves of electric field square versus induced birefringence, which were subjected to curve fitting, in accordance with Equation (2) (Figure 7). After the curve fitting process, available values of physical parameters were achieved: Δ*n_max_* = 0.028, *E_sat_* = 5.4 V·μm^−1^ for the H-PSILC-16.8 cell, and *E_sat_* = 9.6 V·μm^−1^ for the H-PSILC-FS cell. Simultaneously, the Kerr constant is available via the equation, *K = Δn_max_/(λE_sat_^2^)*, so that the Kerr constants were 4.55 nm·V^−2^ in H-PSILC-16.8 cell and 1.44 nm·V^−2^ in H-PSILC-FS cell, respectively. The Kerr constant in H-PSILC-FS cell is larger than the typical values of Kerr constants shown in other references—about 0.2–2 nm·V^−2^ [30,31,32]; our results were possibly improved by the FS3100 dopant.

(2) $$\Delta n_{ind}(E)=\Delta n_{\max}\left[1-\exp\left(-{\left(E/E_{S}\right)}^{2}\right)\right]$$

(3) $$\Delta n_{\max}=\xi \cdot (n_{o}-n_{eff})=\xi \cdot \left(n_{e}-n_{o}\right)/3$$

The maximum birefringence of optically isotropic LCs occurs when all LC directors are parallel to the electric field. These behaviors can be expressed and described by Equation (3). In Equation (3), the ξ is the LC concentration in the cells because other ingredients existing in the cell do not provide refractive index change when electric voltage is applied, and *n_eff_* is the effective refractive index at zero voltage. The calculated ξ value is about ~41% according to Equation (3), which is smaller than the real percentage of HTG LCs: ~65%. This is attributed to the fact that LCs embedded near the polymer networks have difficultly reorienting with respect to the applied electric fields due to strong constraints. To further improve Kerr constants and induced maximum birefringence, some approaches can be efficient, including using optimal percentage ratios between the co-polymer and anisotropic polymer dopant, using LCs with larger birefringence (*Δn*), and using optimal photo-polymerization processes, such as exposure power intensity, exposure time, and process temperature.

## 5. Conclusions

Phase modulators based on PSILC cells are fabricated by means of holographic exposure processes at 120 °C, a temperature which is much higher than the transition temperature of the LC mixture in this study. As a result, fabricated phase modulators show good electro-optical performances, including optical isotropy, low light scattering in the electric off state, and fast optical response times. Surely, S811 chiral dopant plays a key role in improving optical transmittance and optical isotropy. PSILC cells, which were used to fabricate a polarization-independent phase modulator, show the capability for π phase modulation and operation voltage reduction via the FS3100 dopant. If no consideration is given to dielectric breakdown in cells at higher applied voltages, larger phase modulations in the fabricated phase modulators will be obtained. Optimal phase modulators showed fast optical response times (0.11 ms rising time and 1.32 ms falling time) at 100 V_rms_ with π phase modulation. In addition, the extended Kerr effect was used to analyze the induced optical birefringence of PSILC cells in the electric on state. The Kerr constant of an optimal PSILC phase modulator is 4.55 nm·V^−2^. The maxima of induced birefringence and Kerr constant can be improved by means of larger cell gaps and co-polymer dopant [11,20,43]. The proposed polarization-independent phase modulator has a high potential to facilitate the creation of a polarization-independent LC lens array with tunable focal lengths and fast optical response time [61], which are also suitable optical components to be used in integral image systems and augmented reality applications [62].

## Figures and Tables

**Table 1:** Experimental cells with various ratios of ingredients in LC mixtures for processing holographic exposure.

Label of PSILC ^1^ Cells	HTG (wt %)	S811 (wt %)	FS3100 (wt %)	NVP (wt %)	RM257 (wt %)	H-Nu-Blue-640 (wt %)	Borate-V (wt %)
PSILC-0	84	0	0	2	13.5	0.25	0.25
PSILC-4.8	79.2	4.8	0	2	13.5	0.25	0.25
PSILC-8.8	75.2	8.8	0	2	13.5	0.25	0.25
PSILC-12.8	71.2	12.8	0	2	13.5	0.25	0.25
PSILC-16.8	67.2	16.8	0	2	13.5	0.25	0.25
PSILC-FS	65.2	16.8	2	2	13.5	0.25	0.25

^1^ PSILC means an abbreviation of polymer-stabilized isotropic liquid crystal.
